# Covariation of Pluripotency Markers and Biomechanical Properties in Mouse Embryonic Stem Cells

**DOI:** 10.3389/fcell.2022.858884

**Published:** 2022-05-16

**Authors:** Oliver Brookes, Stephen D. Thorpe, Olga Rigby Evans, Michael C. Keeling, David A. Lee

**Affiliations:** ^1^ School of Engineering and Materials Science, Queen Mary University of London, London, United Kingdom; ^2^ UCD School of Medicine, UCD Conway Institute of Biomolecular and Biomedical Research, University College Dublin, Dublin, Ireland

**Keywords:** mouse embryonic stem cells, pluripotency marker, cell stiffness, cell adhesion, cell morphology

## Abstract

Pluripotent cells are subject to much interest as a source of differentiated cellular material for research models, regenerative medical therapies and novel applications such as lab-cultured meat. Greater understanding of the pluripotent state and control over its differentiation is therefore desirable. The role of biomechanical properties in directing cell fate and cell behavior has been increasingly well described in recent years. However, many of the mechanisms which control cell morphology and mechanical properties in somatic cells are absent from pluripotent cells. We leveraged naturally occurring variation in biomechanical properties and expression of pluripotency genes in murine ESCs to investigate the relationship between these parameters. We observed considerable variation in a Rex1-GFP expression reporter line and found that this variation showed no apparent correlation to cell spreading morphology as determined by circularity, Feret ratio, phase contrast brightness or cell spread area, either on a parameter-by-parameter basis, or when evaluated using a combined metric derived by principal component analysis from the four individual criteria. We further confirmed that cell volume does not co-vary with Rex1-GFP expression. Interestingly, we did find that a subpopulation of cells that were readily detached by gentle agitation collectively exhibited higher expression of *Nanog*, and reduced *LmnA* expression, suggesting that elevated pluripotency gene expression may correlate with reduced adhesion to the substrate. Furthermore, atomic force microscopy and quantitative fluorescent imaging revealed a connection between cell stiffness and Rex1-GFP reporter expression. Cells expressing high levels of Rex1-GFP are consistently of a relatively low stiffness, while cells with low levels of Rex1-GFP tend toward higher stiffness values. These observations indicate some interaction between pluripotency gene expression and biomechanical properties, but also support a strong role for other interactions between the cell culture regime and cellular biomechanical properties, occurring independently of the core transcriptional network that supports pluripotency.

## Introduction

In recent years, considerable progress has been made in the development of technologies that utilize pluripotent cells from humans or other mammals. Increasingly, pluripotent stem cells are being utilized as a source of material for regenerative medicine, cellular models of tissue and disease (Maffioletti et al., 2018), and for lab cultured meat products (Reiss et al., 2021). Recently, our understanding of the complexity of the pluripotent state has developed, and although the core role of transcription factors such as Oct3/4 and Nanog have been understood for some years, it is now understood that other transcription factors, such as Rex1, transition through expression states before pluripotent cells commit to differentiation. Most notably, there are pronounced differences between pluripotent cells maintained in different culture media. In mice, embryonic stem cells (ESCs) can be maintained by the addition of leukemia inhibitory factor (LIF) to the medium (Smith et al., 1988; Williams et al., 1988). LIF ultimately activates signal transducer and activator of transcription 3 (STAT3), a transcription factor which upregulates genes associated with self-renewal, including Nanog, and downregulates genes required for differentiation (Hirai et al., 2011). It was later found that combinations of small molecule inhibitors could be used to block signaling associated with early differentiation, such as mitogen-activated protein kinase (MEK; MAPK/ERK kinase 1/2) and glycogen synthase kinase-3β (GSK-3β), establishing a stable phenotype in which expression of pluripotency-associated genes is further enhanced, suggesting that this dual inhibitor “2i” condition may constitute a more fundamental state of pluripotency (Ying et al., 2008). There are noticeable differences in morphology, gene expression and adhesion between the two conditions, and importantly, ESCs can be transitioned reversibly between 2i and LIF medium, changing expression and phenotype accordingly. While the variations in pluripotency state between populations of ESCs maintained in different media conditions have been described in a number of studies, the cellular heterogeneity in pluripotency within a population of cells maintained in the same medium is less well understood.

There are known to be differences in the biomechanical properties of cells that relate to their pluripotency and/or differentiation state. In general, pluripotent cells are softer than most somatic cell types. ESCs typically exhibit stiffness (Young’s modulus) in the range of 0.1–0.5 kPa (Poh et al., 2010; Pagliara et al., 2014), in large part due to the absence of cytoskeletal and nuclear structures which are seen in differentiated cells. Individual pluripotent cells express actin at a low level compared to differentiated cell types (Boraas et al., 2016), and ESCs lack the perinuclear actin (Khatau et al., 2012) or stress fibers (Evans et al., 2009) seen in differentiated cells. The nucleus itself also contributes heavily to cellular biomechanical properties in pluripotent cells, as it occupies a large volume fraction, with proportionally less surrounding cytoplasm in comparison to somatic cells. Many features that typically contribute to nuclear rigidity in somatic cells are reduced or absent in ESCs: chromatin is largely decondensed and can flow under strain (Pajerowski et al., 2007), the lamina and nucleoskeleton contain very little A-type lamin and deform readily (Eckersley-Maslin et al., 2013), and the perinuclear actin that facilitates control of nuclear shape in somatic cells is absent (Khatau et al., 2009). In the naïve state, chromatin is heavily condensed, making the cell and nucleus stiffer, but as pluripotent cells become primed for differentiation, there is a reduction in chromatin condensation resulting in a more pliant nucleus (Chalut et al., 2012). During differentiation cell and nuclear stiffness increases. All these characteristics, in combination, make the primed ESC nucleus softer and more prone to viscous deformation compared to both the naïve and differentiating states. This deformability may favor cell survival during migration (Harada et al., 2014), or facilitate the rapid cell cycle characteristic of pluripotent cells (Becker et al., 2006).

Nonetheless, there is variation between individual pluripotent cells, and mESCs cultured in LIF/serum typically exhibit a mixture of rounded colonies and spread, stellate cells with short processes extending out from the cell perimeter (Blancas et al., 2011). Heterogeneity has also been reported in the expression of pluripotency genes (Chambers et al., 2007; Singh et al., 2007), epigenetic modifications and phenotypic characteristics (Wray et al., 2010; Singer et al., 2014). We leveraged this heterogeneity in naïve ESCs to explore how biomechanical properties might correlate with differences in underlying gene and protein expression. By investigating trends within untreated populations at the level of individual cells, this article complements other recent studies which take a similar phenomenological approach (Lin et al., 2021), as well as traditional experimental works which compare the response of whole populations of cells to applied stimuli.

## Materials and Methods

### Cell Culture and Reagents

The pluripotent reporter line Rex1-GFPd2 was obtained as a gift from Kevin Chalut, University of Cambridge (Nagy et al., 1993; Pagliara et al., 2014). ES-E14TG2a embryonic stem cells were purchased from the European Collection of Authenticated Cell Cultures (ECACC, 08021401, RRID:CVCL_9108) (Hooper et al., 1987).

Basal medium, which was used for routine culture and upon which recipe other media were based, consisted of high-glucose Dulbecco’s modified Eagle’s medium (DMEM; Gibco 61965-026), 10% ESC-qualified fetal bovine serum (FBS; Gibco 16141-079), 1 × non-essential amino acid solution (Gibco 11140-035), 100 U ml^−1^ penicillin, 100 μg ml−1 streptomycin (Sigma-Aldrich P4333), and 1 mM sodium pyruvate (Sigma-Aldrich S8636). For ESC standard culture medium (ESCM), 1,000 U ml^−1^ LIF (Sigma-Aldrich ESGRO) and 100 µM 2-mercaptoethanol (Sigma-Aldrich M7522) were added to basal medium immediately prior to use. 2i medium was prepared from basal medium in which FBS was replaced with 10% knockout serum replacement (KSR, Invitrogen 10820-028), 5 μl ml^−1^ N2 (Invitrogen 17502048), 5 μl ml^−1^ B27 (Invitrogen 17504-044) and 25 μg ml^−1^ bovine serum albumin (BSA; Sigma-Aldrich A9418). LIF is technically dispensable in 2i medium (Ying et al., 2008), although there is evidence that addition of LIF to 2i medium enhances efficiency of clonogenic culture and establishment of new ESC lines (Blair et al., 2011). For this work, LIF was not included in 2i medium unless explicitly stated.

All cells were incubated in a humidified environment at 37°C, 5% CO_2_. For routine culture, passages were made every 48 h. Adherent cells were enzymatically released by incubation with 200–500 μl cm^−2^ of Accutase solution (Sigma-Aldrich, A6964) at 37°C for 2 min and dissociated to a single cell suspension. Cell density was estimated using a Neubauer hemocytometer and a fraction of the suspension was seeded to new flasks at 1×10^4^ cells cm^−2^ to resume culture.

### Immunofluorescent Staining and Imaging

Cells were cultured on imaging dishes and fixed for 10 min at room temperature in 4% paraformaldehyde (PFA) in PBS. After fixation, cells were washed in PBS and permeabilized in a solution of 0.5% Triton X-100 (Sigma-Aldrich T8787) in PBS with 1% BSA for 2 min, followed by three washes in PBS at room temperature. Permeabilized cells were blocked using 4% goat serum (Sigma-Aldrich G9023) in PBS at room temperature for 1 h, or overnight at 4°C. Following this, the imaging dish cover-glass was removed with forceps and washed in PBS. Primary antibody as indicated in Table 1 was diluted in PBS was applied to the sample and incubated overnight at 4°C in a humidified chamber. The sample was washed three times in PBS to remove unbound primary antibody, and a corresponding secondary antibody was applied and incubated for 1 h at room temperature. Before mounting slides, coverslips were washed three times in PBS. Finally, the sample was washed briefly in distilled water to remove PBS, and lowered, face down, onto a 3 µl drop of mounting medium (Prolong Diamond, Molecular Probes 36961) on a glass slide.

**TABLE 1:** Antibodies used in experiments. conj.: conjugated.

Target	Supplier	Cat. No	RRID	Details and dilution
Oct3/4	BD biosciences	560329	AB_1645318	Mouse monoclonal, clone 40/Oct-3, conj. AlexaFluor 647; 1:100
Nanog	Abcam	ab80892	AB_2150114	Rabbit polyclonal, IgG 1:200
SSEA1	BD biosciences	560172	AB_1645310	Mouse monoclonal, clone MC480, conj. AlexaFluor 488; 1:100
SSEA1	BD biosciences	560119	AB_1645314	Mouse monoclonal, clone MC480, conj. AlexaFluor 555; 1:100
Rabbit IgG	Life Tech	A31572	AB_162543	Donkey polyclonal, conj. AlexaFluor 555; 1:1,000

Routine phase contrast microscopy was conducted on a Leica DMIL microscope. Epifluorescence microscopy of fixed specimens was carried out with a Leica DMI 4000B microscope with Leica A4, N3, TX2, CY5 and L5 filter sets. Epifluorescence microscopy of live cells was carried out on an EtaLuma Lumascope 710, installed within an incubator (37°C, 5% CO_2_), using the Lumaview software supplied with the microscope. For live-imaging, Rex1-GFP cells were cultured in ESCM in which the phenol red-containing DMEM had been substituted for Fluorobrite DMEM (Gibco A1896702). Confocal microscopy used a Zeiss LSM 710 ELYRA microscope, fitted with 408, 488, 547, and 633 nm diode lasers. Images were captured using ×20 and ×63 objectives. This was used in conjunction with the fitted INU GM8000 environmental control and environment chamber for live imaging of cells.

### Image Analysis

Image analysis was carried out using ImageJ. Phase contrast images were manually segmented to retrieve cell outlines. For morphological description, cell outlines were measured in terms of area, mean brightness of the original phase contrast image, Feret’s ratio (the ratio between the maximum and minimum caliper diameter of the shape), and circularity (see equation below):

$$4\pi \times \;\frac{\left[Area\right]}{{\left[Perimeter\right]}^{2}}$$

Fluorescent images were captured at 16-bit depth. Background correction consisted of subtraction of a 100-pixel Gaussian filtered copy of the image from the original. This step removes brightness gradients and large-scale illumination artefacts from images while preserving fine detail. In experiments where manually segmented outlines were available, these were used to collect the integrated density (the sum of the pixel values within the outline). In timelapse experiments, cell outlines were produced using the ImageJ default thresholding method.

### Gene Expression Analysis

RNA was extracted with the RNeasy mini kit (Qiagen) following the manufacturer’s instructions. Following extraction, RNA concentration was measured by spectrophotometry (Nanodrop). The Quantitect reverse transcription (RT) kit (Qiagen) was used to transcribe 1 μg isolated RNA to cDNA.

PCR was carried out with TaqMan Fast Advanced master mix (ThermoFisher Scientific 4444963) and TaqMan primer sets (Thermo Fisher Scientific) as listed in Table 2. Negative controls included a no-RT control using the original RNA extraction as a template and a no-template control using water in place of a template. Quantitative PCR was performed in 10 µl reactions in a QuantStudio 7 thermocycler (ThermoFisher Scientific). The QuantStudio software was used to identify threshold-crossing values (Ct). The ∆∆Ct method is then used to estimate relative changes in expression level with TATA-box binding protein (*TBP1*) used as an endogenous control.

**TABLE 2:** TaqMan gene expression assays used for PCR.

Gene symbol	Gene name	Assay ID	Accession no	Amplicon size (bp)	Function
*TBP*	TATA box binding protein	Mm01277042_m1	NM_013684.3	65	Endogenous control
*POU5F1*	Oct3/4	Mm03053917_g1	NM_013633.3	139	Stem-ness markers
*Nanog*	Nanog homeobox	Mm02019550_s1	NM_028016.3	145	
*Rex1*	RNA exonuclease 1	Mm00617735_m1	NM_025852.3	109	
*LmnA*	Lamin A	Mm00497783_m1	NM_019390.3	147	Nuclear envelope components

### Atomic Force Microscopy

AFM measurements were made using a NanoWizard 4 AFM (JPK instruments) mounted on a Zeiss microscope. Newly mounted cantilevers were calibrated by measuring deflection on the glass or plastic substrate. Quantitative imaging (QI) and force spectroscopy measurements of cells were made using a pyramidal (40°) silicon tip on a soft cantilever with a nominal spring constant of 0.03 N m^−1^ (µMasch HQ:CSC38/NO AL, cantilever B). Force spectroscopy measurements were made by indenting at 1 μm s^−1^ up to a cut-off force of 1 nN. QI measurements used similar settings, but with a faster extend speed of 100 μm s^−1^. Analysis of AFM data was carried out using the JPK data processing software (version spm-6.1.22). QI scans were fitted to a Hertz-Sneddon model to estimate Young’s modulus and height values. Curves were corrected for offset and tilt on the basis of the last 15% of the extension curve, and values were calculated assuming a quadratic pyramidal tip with a half-angle to edge of 40°.

### Statistical Methods

All statistical analysis was conducted using Minitab 17 and 18. Significant differences on graphs and tables are indicated using the following *p*-value thresholds: *p ≤ 0.05, **p ≤ 0.01, ***p ≤ 0.001. Pearson’s product moment method was used to calculate correlations, where R is correlation strength and P is statistical significance. For comparisons between categories, data were tested for normality using the Anderson-Darling test and heteroscedasticity using the test for equal variances. Where datasets followed a parametric distribution, experimental groups were compared using two-sided two-sample t-tests. In the case of multiple experimental groups, non-parametric data were transformed to fit a parametric distribution using a Box–Cox transformation prior to analysis of variance and pairwise comparisons. The relationship between cell stiffness and Rex1-GFP integrated density was assessed using linear regression with log transformed Rex1-GFP.

## Results

### Morphological and Genetic Heterogeneity in Pluripotent Cell Populations

**FIGURE 1:**
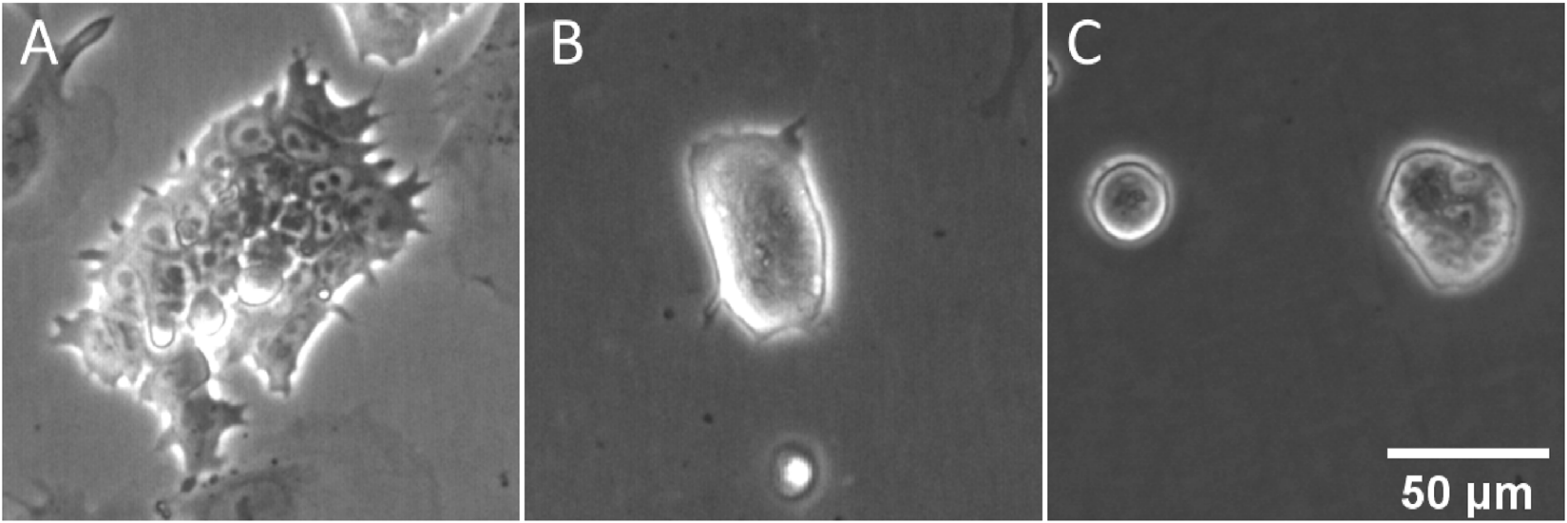
Morphological heterogeneity in ESC colonies maintained with LIF and FBS. Phase contrast images were collected during routine culture of E14TG2a ESCs in both LIF/FBS medium **(A,B)** and 2i/KSR medium **(C)**. In the LIF medium, some colonies can be seen to adopt a compact morphology **(A)**, while other colonies adopt a flattened, pseudo-epithelial morphology **(B)**. In 2i/KSR, cells grow as compact, almost spherical colonies **(C)**.

ESCs grown in culture medium containing serum and LIF exhibit a wide range of colony and single cell morphologies (Supplementary Figure S1). Some colonies in E14TG2a cultures comprise monolayers of small, flat, tightly packed cells (Figure 1A). The nuclei of these cells are visually prominent and can be seen to contain one to three dark subnuclear features, probably nucleoli. In other cases, cells are found as small colonies exhibiting a highly rounded morphology, with steep sides and little visible interaction with the underlying substrate (Figure 1B). In these rounded colonies the nuclei are much less visible. These morphological archetypes represent the extrema of a spectrum of colony morphologies, rather than definitive and separable sub-populations. We can contrast the morphological variety of LIF-maintained ESCs to the marked homogeneity of ESCs grown in 2i medium. These cells display a uniform colony morphology (Figure 1C) similar to the rounder colonies seen in LIF culture. Consistent with these interpretations of adhesion based on morphology, it is noted that 2i ESC colonies become detached from the substrate more easily than LIF ESC colonies, indicative of weak cell-surface interactions. Nuclei are less prominent than in the flattened colonies of LIF, but can be seen in some larger colonies, and where visible appear similar in size and structure to their LIF counterparts.

**FIGURE 2:**
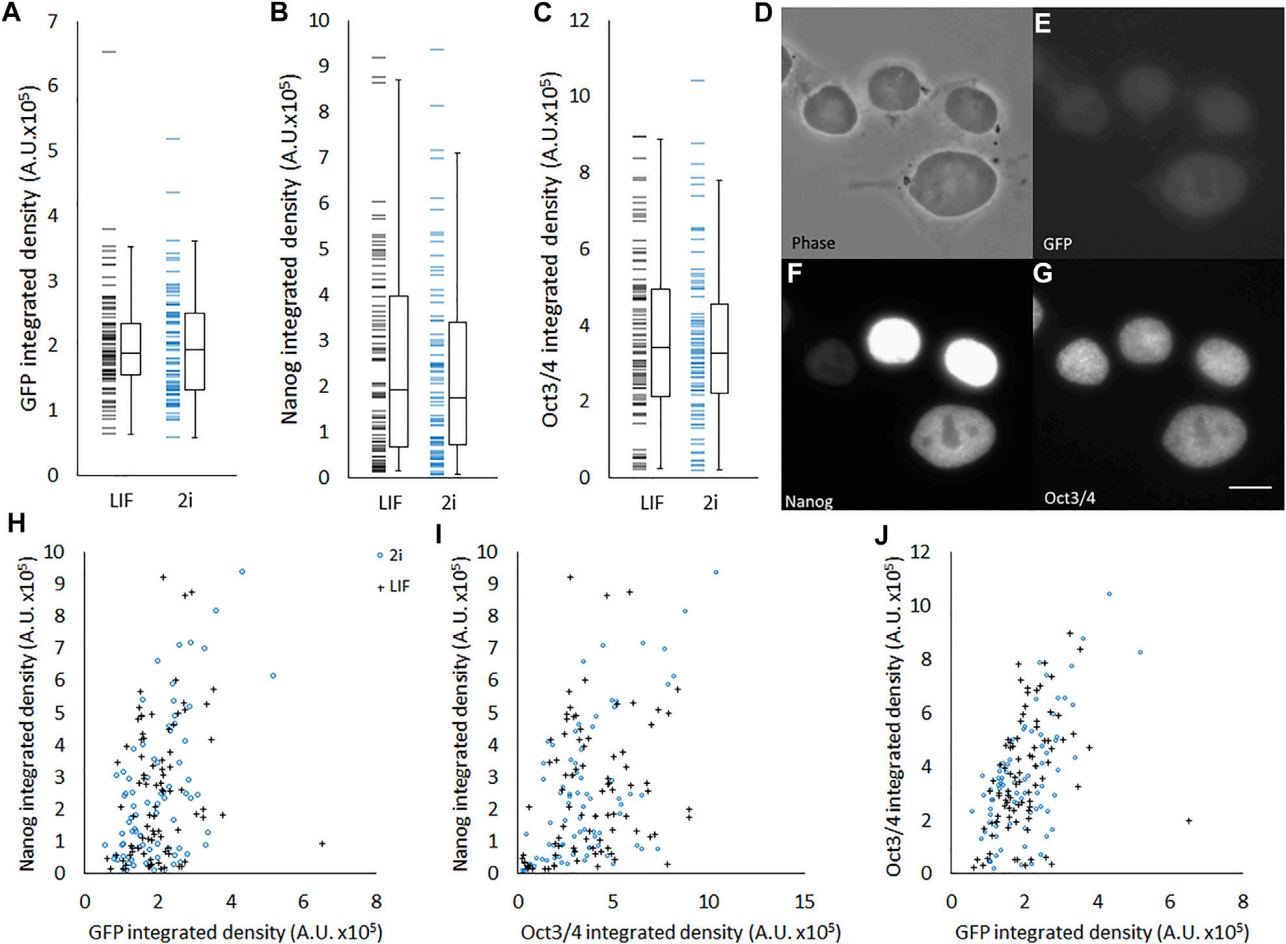
Heterogeneous abundance of Rex1, Oct4, and Nanog in ESCs. Quantification of 3-colour fluorescence from individual Rex1-GFPd2 cells immunostained for Nanog and Oct4. Individual value plots of Rex1-GFP intensity **(A)**, Nanog staining intensity **(B)** and Oct3/4 staining intensity **(C)** all reveal considerable variance across the population. It is not possible in these data to see differences between cells cultured in 2i (*n* = 80) or LIF (*n* = 85). Boxes represent median and interquartile range, with whiskers extending to 1.5× interquartile range or the max/min data points. Representative images **(D–G)** are included to relate brightness of objects to the arbitrary units used in this figure. When plotted against each other **(H–J)** weak correlations are visible. These correlations are quantified in Table 3. Scale bar for D–G: 5 µm.

We hypothesized that pluripotency state was related to morphological characteristics, and therefore that there would be observable correlations between pluripotency gene expression and morphology within heterogenous populations. To verify that this genetic heterogeneity was also observable in our hands, we obtained a *Rex1*-GFP reporter line, Rex1GFPd2, and performed immunofluorescent labelling of Nanog and Oct4 (Figure 2). All three pluripotency-associated transcription factors exhibit a heterogenous pattern of abundance, as reflected in the continuous broad distribution seen across the population for each factor (Figures 2A–C), and this extended to heterogeneity between cells within individual colonies (Figures 2D–G). There is also a significant positive correlation between the strength of the fluorescence attributable to each factor in the immunofluorescence images, as shown in Figures 2H–J and further quantified in Table 3. The strongest correlation was observed between Oct4 and *Rex1*-GFP, which supports the use of Rex1GFPd2 as a general indicator of pluripotency, given the central role of Oct4 in maintaining the pluripotent state. Contrary to other work that has been published indicating that 2i cells exist in a homogeneous, ‘ground state’ of pluripotency (Singh et al., 2007; Ying et al., 2008), we observed no significant difference between LIF and 2i conditions in terms of the distributions of Nanog, Oct4 or Rex1 dependent GFP integrated density. The tight nuclear staining we observed for Oct4 and Nanog is consistent with expectations for localization of these transcription factors.

**TABLE 3:** Correlations between fluorescent staining/reporter intensities of major pluripotency associated factors.

	Rex1 (GFP)	Nanog (Alexa 555)
Nanog (Alexa 555)	R = 0.376	-
Oct4 (Alexa 647)	R = 0.511	R = 0.433

Rex1-GFP cells were co-stained with anti-Oct4 and anti-Nanog antibodies. The correlation between the integrated densities of Nanog staining and Rex1-dependent GFP fluorescence is weak, as is the correlation between Nanog and Oct4 staining. The strongest correlation was seen between Oct4 and Rex1. Pearson’s correlation, *n* = 165, all *p* values < 0.001.

As it has been observed that Rex1 may vary during the cell cycle (Coronado et al., 2013), it is necessary to gauge the extent to which *Rex1*-dependent GFP fluorescence is dependent on cell cycle. We have estimated cell doubling time to be around 17 h, based on cell counts over a 72 h period (Supplementary Figure S2). In general, GFP fluorescence increases in a cell over time, with some cells showing the reverse trend (arrowheads, Supplementary Figure S3A). In many cells the fluorescence varies considerably over time, but there is no overlap between the cells with the highest expression of GFP and those with the lowest. In terms of morphology, the highest and lowest expressing cells appear qualitatively to have different morphology (inset images, Supplementary Figure S3A). However there is near-complete overlap in spread area over time for the measured cells, and there is no trend of higher GFP expressors having a different area from the lowest (Supplementary Figure S3B).

### Interaction Between Rex1 Expression Level and Cell Morphology

**FIGURE 3:**
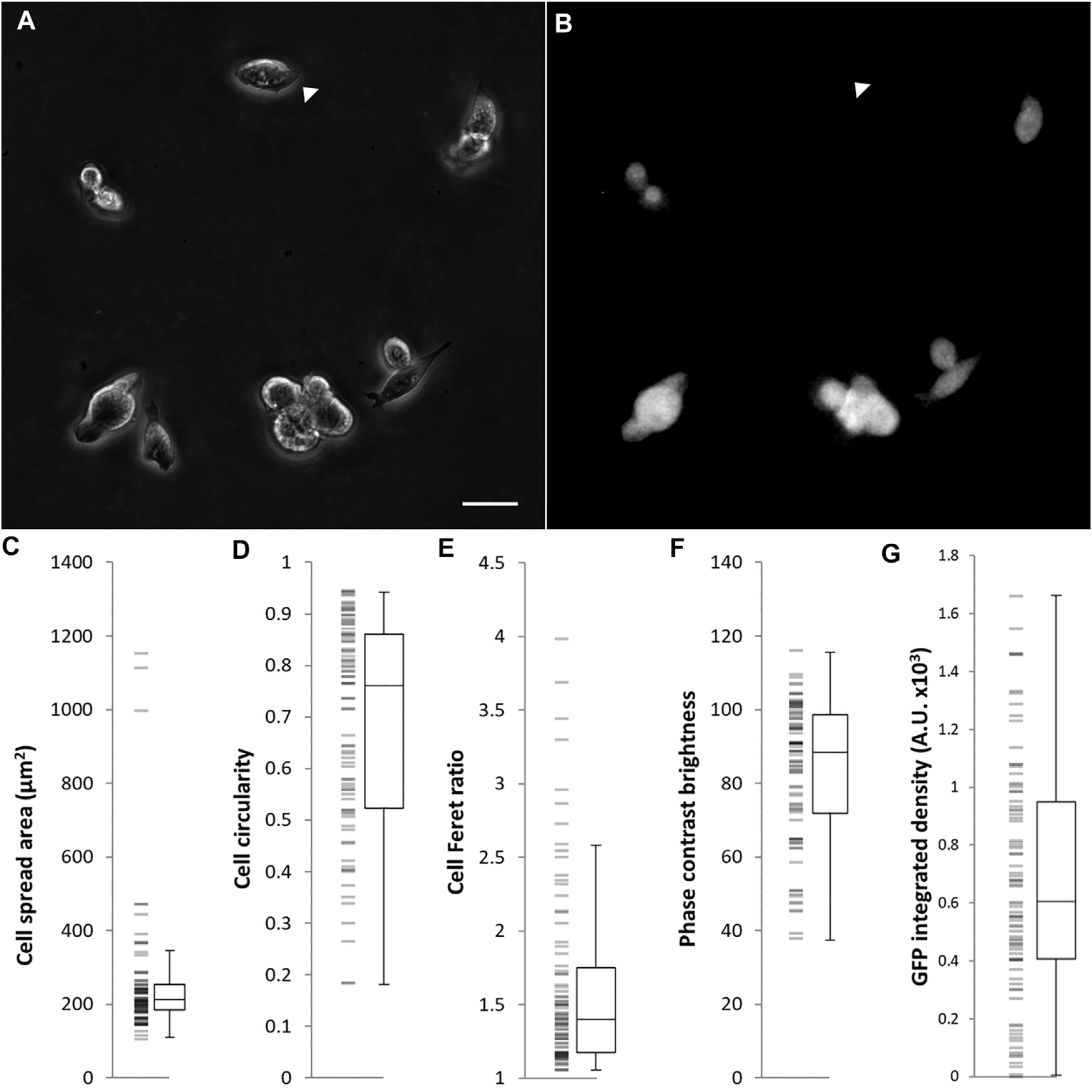
Morphological characterization of Rex1-GFP mESCs growing in LIF/FBS medium on gelatin. Rex1-GFP cells were seeded on gelatin-coated imaging dishes, cultured for 6 h and imaged. As can be seen in the phase contrast image **(A)**, a range of morphologies were observed in this population. Specific parameters of single cells are quantified in **(C–F)**. As expected, the Rex1-GFP reporter is expressed heterogeneously **(B)**, arrowheads indicate Rex1-GFP negative cell. Rex1-GFP is quantified in **(G)**. Scale bar: 25 µm. Boxes represent median and interquartile range, with whiskers extending to 1.5× interquartile range or the max/min data points. *n* = 84.

**FIGURE 4:**
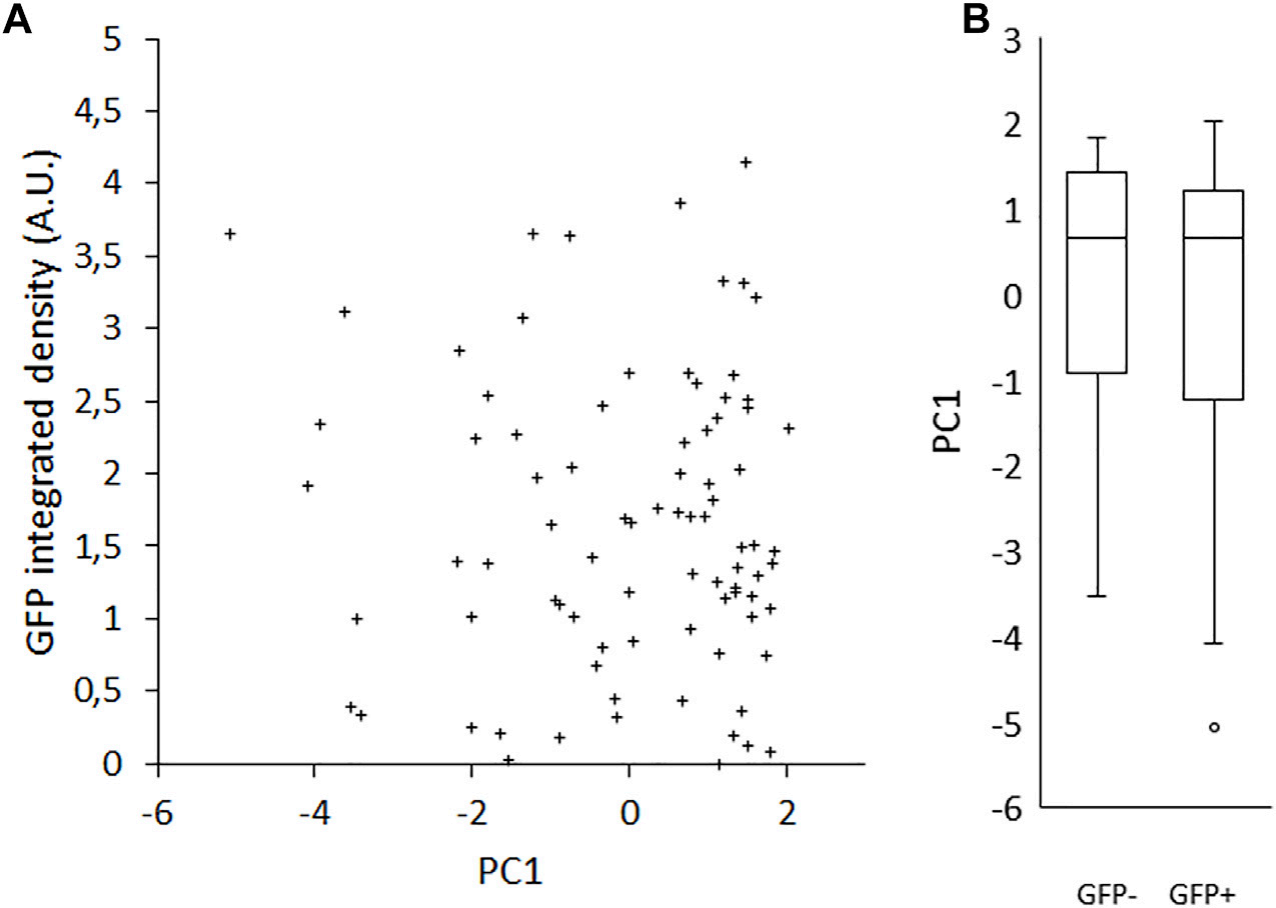
Effect of Rex1-GFP expression on morphological index (PC1). Rex1-GFPd2 cells were cultured in LIF/FBS medium for 6 h and imaged live. Morphological traits were measured from phase contrast images and reduced by principal components analysis to a single metric, PC1, compared here to GFP fluorescence integrated density from the same cells. There is no correlation between PC1 and the intensity of GFP expression from the Rex1 promoter (**A**, *R* = −0.069). This is also reflected in the individual value plot of sub-median (−) vs. super-median (+) GFP integrated density against PC1 **(B)**. There is no apparent difference in the distribution of the two groups, which implies that there is no relationship between Rex1 expression and cell morphology. Median GFP I.D. = 1.50023 A.U., GFP+ and GFP- denote cells above or below median, respectively. Boxes represent median and interquartile range, with whiskers extending to 1.5× interquartile range or the max/min data points. *n* = 84.

Because interactions between cells within a colony may influence the morphology of cells, we seeded single cells on gelatin-coated imaging dishes, in LIF/FBS medium. To avoid potential disruption of cell morphology or detachment of cells during processes of fixation, staining and mounting, we imaged the cells live after 6 h of culture. In this time, many of the cells had not divided and were visible as single cells. As was the case with colony morphology, we observed a variety of cell morphologies, ranging from rounded to spread (Figure 3A). A heterogeneous pattern of *Rex1*-dependent GFP expression was also observed (Figure 3B). Images were collected and single cells were manually segmented in ImageJ and parameterized according to four metrics: cell spread area, cell circularity, cell Feret ratio (the ratio between the maximum and minimum dimensions of the convex hull of the cell outline) and mean cell brightness (Figures 3C–F). For each cell outline, the total cell GFP fluorescence was also quantified (GFP integrated density, Figure 3G). The cell morphology data are not normally distributed (Anderson-Darling, *p* < 0.005). Principal component analysis was used to reduce the cell shape variables to a single parameter that describes the rounded-ness/spread-ness of cells. The first principal component (PC1 hereafter) accounts for about 70% of the variability within the dataset (Supplementary Figure S4A), and correlates well with each of the original variables (Supplementary Figures S4B–E), suggesting that each variable contributes meaningfully to the variance of the whole data space. Indeed, the loading of each variable in PC1 is roughly equal (Table 4, title row). GFP integrated density was not correlated with morphology, either with the combined metric PC1 (Figure 4A) or with any individual measured parameter (Supplementary Figure S5). There was no significant difference between the PC1 characteristic of cells above and below median GFP integrated density (Figure 4B), disproving our hypothesis that cells with a 2i-like morphology exhibit stronger expression of GFP and, therefore, of Rex1.

**TABLE 4:** Correlations between shape characteristics of cells cultured in serum/LIF medium.

Variable *Coefficient in PC1*	Feret ratio *−0.480*	Circularity *0.521*	Phase brightness *0.527*	Area *−0.470*
Area	R = 0.195 *p* = 0.076	R = 0.489 *p* < 0.005	R = *−*0.765 *p* < 0.005	-
Phase Brightness	R = *−*0.669 *p* < 0.005	R = 0.770 *p* < 0.005	-	-
Circularity	R = *−*0.770 *p* < 0.005	-	-	-

Rex1-GFPd2 cells growing on gelatin. Correlations are assessed using Pearson’s *R*, where *R* gives the strength of the correlation from −1 to 1 and p indicates the probability of the null hypothesis that this R value arose by chance. There are strong correlations between the variables used to indicate cell shape, reflecting causal relationships. However, these causal links are not so strong that any two of these variables are mutually redundant.

### Cell Volume Variation is Independent of Rex1 and SSEA1 Expression

**FIGURE 5:**
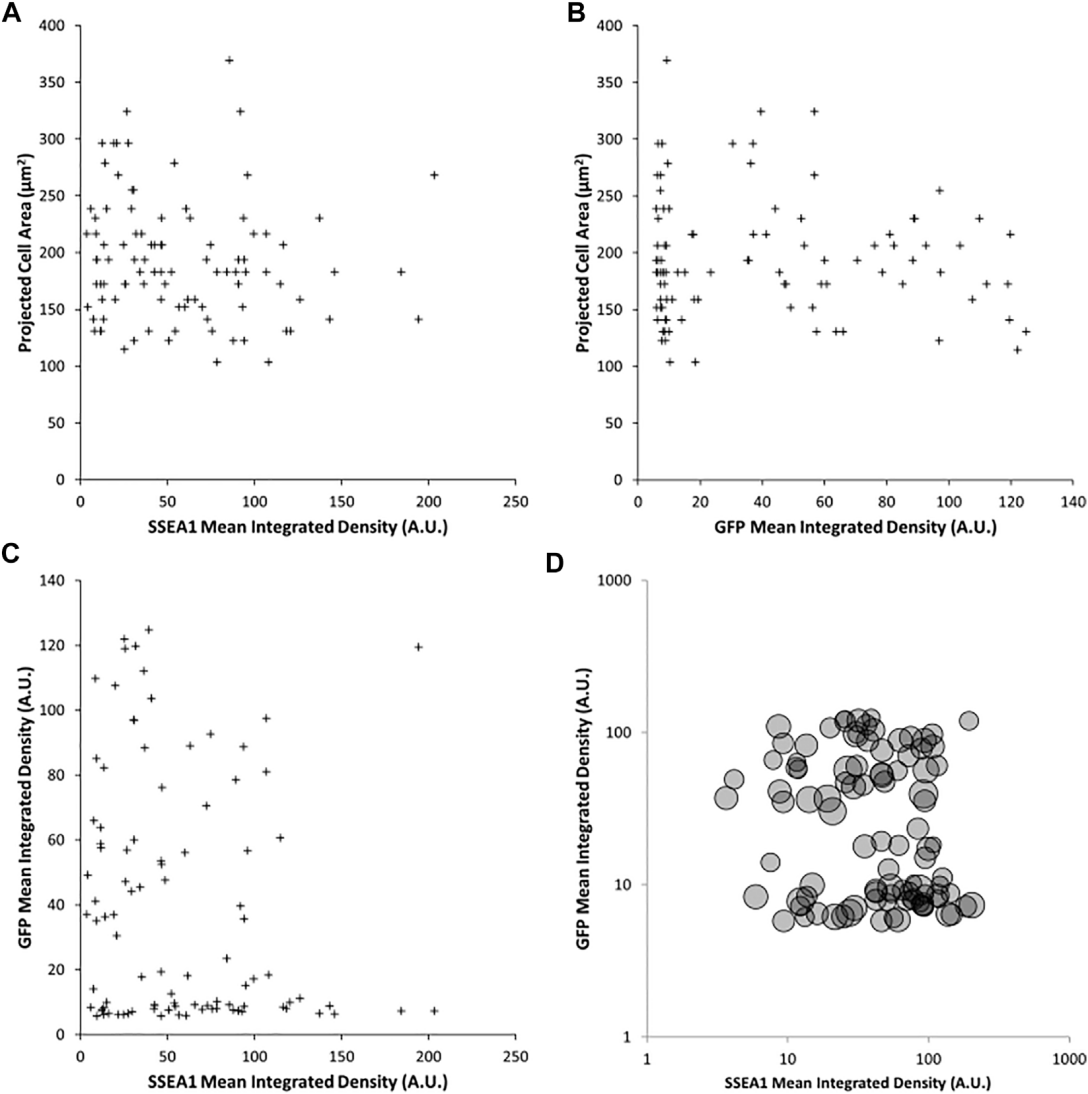
Effect of Rex1 expression and SSEA1 abundance on cell volume. Live Rex1-GFPd2 cells cultured in LIF/FBS were enzymatically dissociated, labelled with an anti-SSEA1 antibody and imaged in suspension in phase contrast and fluorescent modes. Cells in suspension are assumed to adopt a spherical shape, so cell volume can be estimated from projected area. Projected area of detached Rex1GFP cells weakly correlates to SSEA1 abundance [**(A)**, *R* = 0.261, *p* = 0.034], and Rex1-dependent GFP fluorescence [**(B)**, *R* = −0.306, *p* = 0.002]. Surprisingly, there is no apparent correlation between SSEA1 abundance and *Rex1*-dependent GFP expression [**(C)**, *R* = −0.167, *p* = 0.102]. There is no covariance in terms of the effect of these pluripotency markers on cell volume [**(D)**, circle areas correspond to projected cell areas]. *n* = 97, Pearson correlation test.

It is conceivable that the combined effects of cellular properties such as volume, contractility and compliance could co-vary in such a way that spread area remains constant, masking the impact of pluripotent state on the fundamental mechanical properties of the cell. To test the relationship between volume and *Rex1*-GFP expression, cells were enzymatically detached from the substrate and imaged in suspension, in which condition they become approximately spherical, allowing the measurement of projected area as a proxy for cell volume. There is weak correlation between this metric and either *Rex1*-GFP (Figure 5A, *R* = 0.261, *p* = 0.034) or SSEA1 (Figure 5B, *R* = −0.306, *p* = 0.002) integrated density. Interestingly, there also appears to be little to no correlation between *Rex1*-GFP and SSEA1 integrated density (Figure 5C), suggesting that these two commonly used indicators of pluripotency can vary independently of one another in terms of gene expression and/or protein abundance. When detached, it is apparent that *Rex1* reporter fluorescence falls into two distinct ranges when integrated fluorescence is plotted on a logarithmic axis (Figure 5D).

### Interaction Between Adhesion Strength and Pluripotency Gene Expression

**FIGURE 6:**
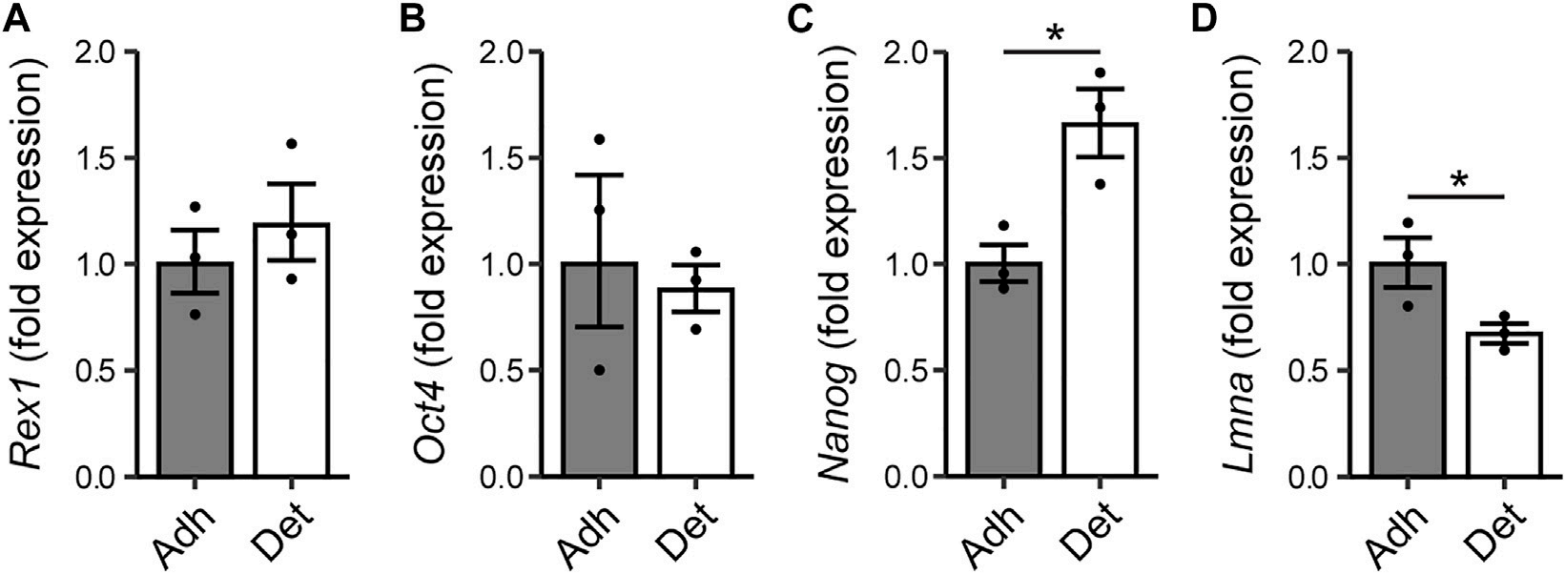
Relationship between adhesion of cells and expression of pluripotency markers. Rex1-GFPd2 cells, cultured in LIF/FBS medium, were subjected to fluid shear 24 h after cell seeding, causing a proportion of the cells to become mechanically detached from the substrate. RNA was extracted separately from detached cells and the remaining adherent cells. Quantitative PCR reveals no difference in expression of *Rex1* or *Oct4* **(A,B)**, but the readily detached cells have higher expression of *Nanog* **(C)**, and reduced expression of lamin A **(D)**. Fold expression is calculated relative to the adherent cell condition. Mean ± s.e.m. with individual values overlaid. *n* = 3, Two-sample *t*-test on ∆∆Ct values, *p < 0.05.

The morphological heterogeneity of ESCs cultured in FBS/LIF is likely the result of differences in substrate adhesion, among other forces. Rounded cells and colonies are weakly attached to the substrate and are readily detached. To investigate the relationship between adhesive strength and pluripotent state, cells were detached mechanically by agitation in PBS, and RNA extracted separately from the adherent and detached subpopulations. The more readily detached cells exhibited a similar expression level of the *Rex1* and *Oct4* (*Pou5f1*) genes to the adherent cell population (Figures 6A,B). However, these readily detached cells had higher levels of transcriptional activity of *Nanog* (Figure 6C), and reduced expression of *LmnA*, which encodes lamin A/C and is used here as a positive indicator of differentiation (Figure 6D). Taken together, these attributes may support the proposition that differences in cell-substrate adhesion are associated with changes in pluripotency, but that this likely occurs after the transition from naïve to primed.

### Relationship Between Cell Stiffness and Rex1-GFP Fluorescence

**FIGURE 7:**
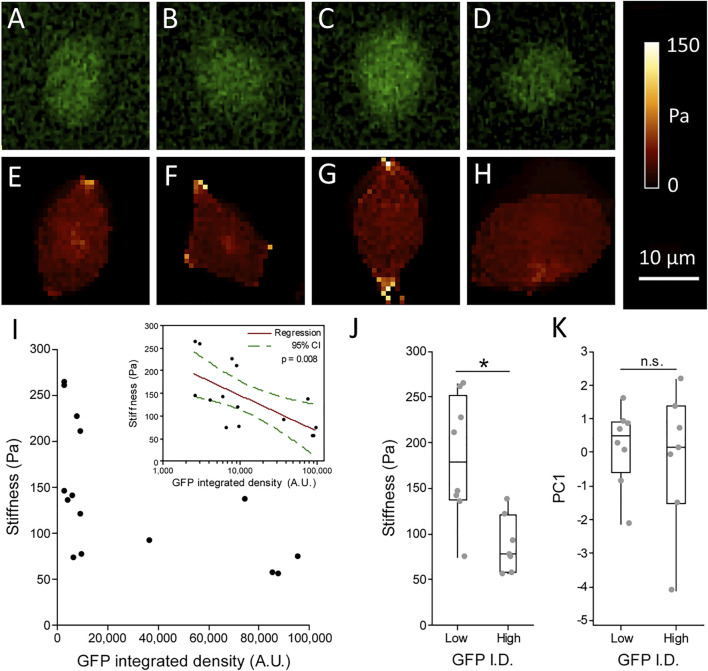
Relationship between Stiffness and Rex1-GFP expression in individual cells. Live Rex1-GFPd2 cells cultured in LIF/FBS were seeded into glass-bottomed imaging dishes and subjected to AFM force spectroscopy combined with fluorescence microscopy, so that GFP integrated density **(A–D)** and stiffness **(E–H)** could be determined on a cell-by-cell basis. Figures **(E–H)** are high resolution maps, while cell stiffness values presented in **(I–J)** are the median of multiple point measurements within individual cells (5 measurements each, *n* = 15 cells, Gavara and Chadwick 2015). (**I**) Stiffness plotted against GFP integrated density (I.D.) revealed all cells stiffer than median (137 Pa) have low GFP intensity, while cells above median brightness (∼8.9 × 10^3^ A.U.) are also soft **(I**, grouped data in **J)**. Inset: Stiffness is negatively associated with GFP expression. *n* = 15, Linear regression with log transformed GFP integrated density, *p* = 0.008. Consistent with prior results, morphology does not seem to be connected to Rex1-GFP expression **(K)**. Boxes represent median and interquartile range, with whiskers extending to 1.5× interquartile range or the max/min data points. *n* = 7–8, Mann-Whitney test, n.s. *p* = 0.862, *p = 0.013.

AFM coupled with epifluorescent microscopy was used to probe individual cells and relate stiffness to Rex1-GFP fluorescence intensity. Phase contrast images were also taken, allowing further comparisons with metrics describing cell morphology. Example images of GFP fluorescence and the corresponding cell stiffness maps can be seen in Figures 7A–H. From the graph of these data (Figure 7I) it appears that while cells can adopt different combinations of high or low stiffness and high or low GFP expression, we did not observe cells with simultaneously high GFP and high stiffness. Accordingly, cells with the highest Rex1-GFP reporter fluorescence all have low stiffness, while the stiffest cells exclusively exhibit low Rex1-GFP reporter activity (Figure 7I and inset). Dividing the population on the basis of GFP integrated density, all cells in the upper median range (GFP high, median value 8,902 A.U.) have stiffness values in the range of 56-137 Pa, while those in the lower median range (GFP low) have stiffness values ranging from 74 to 264 Pa, and are significantly different at the 95% confidence level (Figure 7J). The GFP high cells were not significantly different from the GFP low population with respect to the morphological parameters that were investigated (Figure 7K).

## Discussion

In this study we have examined associations between pluripotent state, morphology and biomechanical properties within the range of variation that is seen in a single population, rather than comparing parallel populations that have been subjected to different treatment as many prior studies do. This has the advantage of avoiding covariant, off-target effects that are induced directly by the treatment regime and are not directly causally related to pluripotency gene expression. To achieve this, we have exploited the heterogeneous variation that exists in mESCs maintained in LIF/FBS medium.

Significant heterogeneity exists within populations of ESCs in LIF/FBS medium, in terms of both morphological phenotype (Figures 1, 3) and pluripotency marker expression (Figure 2). By contrast, cells maintained in 2i/KSR have more consistent cell morphology (Figure 1C), albeit with similar variation in gene expression (Figure 2). On the basis of these observations, we hypothesized that variation in expression of pluripotency markers would be causally linked to morphology, anticipating that changes in cellular and nuclear architecture would occur which drive this. However, contrary to our expectations, we found no correlation between *Rex1*-dependent GFP expression and general cell morphology (Figure 4).

In order to validate Rex1 as a reporter of pluripotency, we have confirmed that Rex1-dependent GFP fluorescence integrated density correlates to the integrated density of fluorescence from immunolabelled Oct4 and Nanog, and that there is nonetheless considerable latitude for independent variation of the three (Figure 2). This is consistent with observations published by others (Faddah et al., 2013), which show correlations between the pluripotency genes and cell-cell variation of pluripotency gene transcript abundance. Nonetheless, as *Rex1*-dependent GFP fluorescence is broadly correlated to abundance of the core pluripotency-associated transcription factors Oct4 and Nanog, it is valid to use it as a general measure of pluripotency gene/protein expression, provided no further interventions have been made which might be reasonably expected to cause differential expression of these various factors. With respect to the known dependence of Rex1 on cell cycle stage (Coronado et al., 2013), time-resolved observation of a randomly selected cohort of cells over a large part of the cell cycle revealed no instances of cells transitioning from the lowest levels of Rex1 expression to the highest or vice versa, supporting the idea that there is some adoption of higher or lower levels of Rex1 by these cells over longer timescales.

The independence of pluripotent state and morphological properties implies that the rounded morphology commonly seen in ESCs or iPSCs in conditions which foster robust pluripotency (e.g., 2i culture) may not arise as a result of “ground state” pluripotency; but rather as a secondary consequence of culture regimes that are typically used to initiate and maintain pluripotency. Differences in spreading morphology could be the direct result of differences in the environment presented to the cell (for instance a low abundance or absence of serum proteins in chemically defined media). Alternatively, they could be the result of changes in intracellular processes (e.g., reduced contractility or actin polymerization) unrelated to pluripotency. This disconnect is surprising, however, in light of other studies in which actin-mediated transfer of cytoskeletal tension to the nucleus was found to support the differentiation of pluripotent cells (Chowdhury et al., 2010; David et al., 2019), suggesting that morphology and pluripotency would be intimately related.

Cells which detach more readily from the surface exhibit higher expression of the pluripotency gene *Nanog*, and reduced expression of *LmnA*, which encodes nuclear envelope proteins considered indicative of differentiation. This observation is consistent with the relatively weak attachment seen in ESCs cultured in 2i medium compared to those maintained with LIF alone, given that the former phenotype is considered closer to the hypothetical “ground state” of pluripotency (Wray et al., 2010). However, the effect was not exhibited with further pluripotency markers, as there was no significant difference in *Oct4* or *Rex1* expression between adherent and detached populations. It should also be noted that detachment of cells may also relate to cell cycle, with cells undergoing mitosis typically being less adherent than at other phases of the cell cycle. This may also relate to the differences in *LmnA* expression, as lamin A is a component of the nucleoskeleton as well as being considered a differentiation marker and therefore may be present at reduced levels as the nuclear membrane is disrupted during M phase.

In terms of directly measured biomechanical properties, there is a significant difference in stiffness between sub-median and super-median Rex1-GFP fluorescence groups, implying that stiffer cells express less *Rex1*, while cells that express abundant *Rex1* are characteristically softer. The existence of soft, low GFP-intensity cells indicates that decline in expression of *Rex1* does not directly mediate a transition to a higher stiffness mode of the cell. However, high expression of *Rex1* may inhibit formation of structures and features integral to cell stiffness, or other pluripotent transcription factors might simultaneously promote *Rex1* and maintain the soft state of the cell. Previous studies have assessed ESC stiffness at the cell and nuclear level in a range of pluripotency states (Pillarisetti et al., 2011; Chalut et al., 2012). These studies have demonstrated that cells in the primed state have the lowest stiffness, when compared to cells in the naïve/ground state or as differentiation progresses. Chalut et al. (2012), in particular, describe the difference between high and low Nanog states, representing naïve and primed states respectively, with cell stiffness greater in the high Nanog/naïve state. These findings may appear to contradict the data in the current study that indicate that the stiffest cells express low levels of the pluripotency marker *Rex1*. However, the LIF medium we have used in our cell stiffness study is not considered to support the naïve/ground state, which is typically maintained using alternative culture conditions and media (including 2i medium). Our data may, therefore, represent heterogeneity in cell stiffness within the primed state of pluripotency. It is, therefore, not unexpected that cells within our study that have the highest Rex1-GFP, representing the primed state, are soft, while cells that are stiffer potentially transitioning from the primed state and towards differentiation have lower expression of GFP.

In conclusion, we have leveraged the native heterogeneity of non-ground-state ESCs to investigate relationships between pluripotency, morphology, and cellular biomechanical properties. We have found evidence that morphological parameters which vary between culture regimes are causally unrelated to pluripotency. Meanwhile, cell stiffness and adhesion strength do appear somewhat dependent on pluripotency gene expression over this range.

## Data Availability

The data that support the findings of this study are available from the corresponding authors upon reasonable request.

## References

[B1] Becker K. A. Ghule P. N. Therrien J. A. Lian J. B. Stein J. L. van Wijnen A. J. (2006). Self-Renewal of Human Embryonic Stem Cells Is Supported by a Shortened G1 Cell Cycle Phase. J. Cell. Physiol. 209, 883–893. 10.1002/jcp.20776 16972248

[B2] Blair K. Wray J. Smith A. (2011). The Liberation of Embryonic Stem Cells. PLoS Genet. 7, e1002019. 10.1371/journal.pgen.1002019 21490948 PMC3072365

[B3] Blancas A. A. Chen C.-S. Stolberg S. McCloskey K. E. (2011). Adhesive Forces in Embryonic Stem Cell Cultures. Cell Adhes. Migr. 5, 472–479. 10.4161/cam.5.6.18270 PMC3277780 22274712

[B4] Boraas L. C. Guidry J. B. Pineda E. T. Ahsan T. (2016). Cytoskeletal Expression and Remodeling in Pluripotent Stem Cells. PLoS One 11, e0145084–16. 10.1371/journal.pone.0145084 26771179 PMC4714815

[B5] Chalut K. J. Höpfler M. Lautenschläger F. Boyde L. Chan C. J. Ekpenyong A. (2012). Chromatin Decondensation and Nuclear Softening Accompany Nanog Downregulation in Embryonic Stem Cells. Biophys. J. 103, 2060–2070. 10.1016/j.bpj.2012.10.015 23200040 PMC3512036

[B6] Chambers I. Silva J. Colby D. Nichols J. Nijmeijer B. Robertson M. (2007). Nanog Safeguards Pluripotency and Mediates Germline Development. Nature 450, 1230–1234. 10.1038/nature06403 18097409

[B7] Chowdhury F. Li Y. Poh Y. C. Yokohama-Tamaki T. Wang N. Tanaka T. S. (2010). Soft Substrates Promote Homogeneous Self-Renewal of Embryonic Stem Cells via Downregulating Cell-Matrix Tractions. PLoS One 5, e15655. 10.1371/journal.pone.0015655 21179449 PMC3001487

[B8] Coronado D. Godet M. Bourillot P.-Y. Tapponnier Y. Bernat A. Petit M. (2013). A Short G1 Phase Is an Intrinsic Determinant of Naïve Embryonic Stem Cell Pluripotency. Stem Cell Res. 10, 118–131. 10.1016/j.scr.2012.10.004 23178806

[B9] David B. G. Fujita H. Yasuda K. Okamoto K. Panina Y. Ichinose J. (2019). Linking Substrate and Nucleus via Actin Cytoskeleton in Pluripotency Maintenance of Mouse Embryonic Stem Cells. Stem Cell Res. 41, 101614. 10.1016/j.scr.2019.101614 31715427

[B10] Eckersley-Maslin M. A. Bergmann J. H. Lazar Z. Spector D. L. (2013). Lamin A/C Is Expressed in Pluripotent Mouse Embryonic Stem Cells. Nucleus 4, 53–60. 10.4161/nucl.23384 23324457 PMC3585028

[B11] Evans N. Minelli C. Minelli C. Gentleman E. LaPointe V. Patankar S. (2009). Substrate Stiffness Affects Early Differentiation Events in Embryonic Stem Cells. eCM 18, 1–14. 10.22203/ecm.v018a01 19768669

[B12] Faddah D. A. Wang H. Cheng A. W. Katz Y. Buganim Y. Jaenisch R. (2013). Single-cell Analysis Reveals that Expression of Nanog Is Biallelic and Equally Variable as that of Other Pluripotency Factors in Mouse Escs. Cell Stem Cell 13, 23–29. 10.1016/j.stem.2013.04.019 23827708 PMC4035816

[B13] Gavara N. Chadwick R. S. (2015). Relationship between Cell Stiffness and Stress Fiber Amount, Assessed by Simultaneous Atomic Force Microscopy and Live-Cell Fluorescence Imaging. Biomech. Model Mechanobiol. 15, 511–523. 10.1007/s10237-015-0706-9 26206449 PMC4869747

[B14] Harada T. Swift J. Irianto J. Shin J.-W. Spinler K. R. Athirasala A. (2014). Nuclear Lamin Stiffness Is a Barrier to 3D Migration, but Softness Can Limit Survival. J. Cell Biol. 204, 669–682. 10.1083/jcb.201308029 24567359 PMC3941057

[B15] Hirai H. Karian P. Kikyo N. (2011). Regulation of Embryonic Stem Cell Self-Renewal and Pluripotency by Leukaemia Inhibitory Factor. Biochem. J. 438, 11–23. 10.1042/bj20102152 21793804 PMC3418323

[B16] Hooper M. Hardy K. Handyside A. Hunter S. Monk M. (1987). HPRT-deficient (Lesch-Nyhan) Mouse Embryos Derived from Germline Colonization by Cultured Cells. Nature 326, 292–295. 10.1038/326292a0 3821905

[B17] Khatau S. B. Hale C. M. Stewart-Hutchinson P. J. Patel M. S. Stewart C. L. Searson P. C. (2009). A Perinuclear Actin Cap Regulates Nuclear Shape. Proc. Natl. Acad. Sci. U.S.A. 106, 19017–19022. 10.1073/pnas.0908686106 19850871 PMC2776434

[B18] Khatau S. B. Kusuma S. Hanjaya-Putra D. Mali P. Cheng L. Lee J. S. (2012). The Differential Formation of the LINC-Mediated Perinuclear Actin Cap in Pluripotent and Somatic Cells. PLoS One 7, e36689–12. 10.1371/journal.pone.0036689 22574215 PMC3344930

[B19] Lin S. C. Loza A. Antrim L. Talbot P. (2021). Video Bioinformatics Analysis of Human Pluripotent Stem Cell Morphology, Quality, and Cellular Dynamics. Stem Cells Transl. Med. 10, 1343–1359. 10.1002/sctm.15-0352 34089307 PMC8380446

[B20] Maffioletti S. M. Sarcar S. Henderson A. B. H. Mannhardt I. Pinton L. Moyle L. A. (2018). Three-Dimensional Human iPSC-Derived Artificial Skeletal Muscles Model Muscular Dystrophies and Enable Multilineage Tissue Engineering. Cell Rep. 23, 899–908. 10.1016/j.celrep.2018.03.091 29669293 PMC5917451

[B21] Nagy A. Rossant J. Nagy R. Abramow-Newerly W. Roder J. C. (1993). Derivation of Completely Cell Culture-Derived Mice from Early-Passage Embryonic Stem Cells. Proc. Natl. Acad. Sci. U.S.A. 90, 8424–8428. 10.1073/pnas.90.18.8424 8378314 PMC47369

[B22] Pagliara S. Franze K. McClain C. R. Wylde G. W. Fisher C. L. Franklin R. J. M. (2014). Auxetic Nuclei in Embryonic Stem Cells Exiting Pluripotency. Nat. Mater 13, 638–644. 10.1038/nmat3943 24747782 PMC4283157

[B23] Pajerowski J. D. Dahl K. N. Zhong F. L. Sammak P. J. Discher D. E. (2007). Physical Plasticity of the Nucleus in Stem Cell Differentiation. Proc. Natl. Acad. Sci. U.S.A. 104, 15619–15624. 10.1073/pnas.0702576104 17893336 PMC2000408

[B24] Pillarisetti A. Desai J. P. Ladjal H. Schiffmacher A. Ferreira A. Keefer C. L. (2011). Mechanical Phenotyping of Mouse Embryonic Stem Cells: Increase in Stiffness with Differentiation. Cell. Reprogr. 13, 371–380. 10.1089/cell.2011.0028 21728815

[B25] Poh Y.-C. Chowdhury F. Tanaka T. S. Wang N. (2010). Embryonic Stem Cells Do Not Stiffen on Rigid Substrates. Biophys. J. 99, L19–L21. 10.1016/j.bpj.2010.04.057 20643049 PMC2905070

[B26] Reiss J. Robertson S. Suzuki M. (2021). Cell Sources for Cultivated Meat: Applications and Considerations throughout the Production Workflow. Int. J. Mol. Sci. 22, 7513. 10.3390/ijms22147513 34299132 PMC8307620

[B27] Singer Z. S. Yong J. Tischler J. Hackett J. A. Altinok A. Surani M. A. (2014). Dynamic Heterogeneity and DNA Methylation in Embryonic Stem Cells. Mol. Cell 55, 319–331. 10.1016/j.molcel.2014.06.029 25038413 PMC4104113

[B28] Singh A. M. Hamazaki T. Hankowski K. E. Terada N. (2007). A Heterogeneous Expression Pattern for Nanog in Embryonic Stem Cells. Stem Cells 25, 2534–2542. 10.1634/stemcells.2007-0126 17615266

[B29] Smith A. G. Heath J. K. Donaldson D. D. Wong G. G. Moreau J. Stahl M. (1988). Inhibition of Pluripotential Embryonic Stem Cell Differentiation by Purified Polypeptides. Nature 336, 688–690. 10.1038/336688a0 3143917

[B30] Williams R. L. Hilton D. J. Pease S. Willson T. A. Stewart C. L. Gearing D. P. (1988). Myeloid Leukaemia Inhibitory Factor Maintains the Developmental Potential of Embryonic Stem Cells. Nature 336, 684–687. 10.1038/336684a0 3143916

[B31] Wray J. Kalkan T. Smith A. G. (2010). The Ground State of Pluripotency. Biochem. Soc. Trans. 38, 1027–1032. 10.1042/bst0381027 20658998

[B32] Ying Q.-L. Wray J. Nichols J. Batlle-Morera L. Doble B. Woodgett J. (2008). The Ground State of Embryonic Stem Cell Self-Renewal. Nature 453, 519–523. 10.1038/nature06968 18497825 PMC5328678

