# Supplementary material for: Covariation of Pluripotency Markers and Biomechanical Properties in Mouse Embryonic Stem Cells
Source: Front Cell Dev Biol. 2022 May 16;10:858884. doi: 10.3389/fcell.2022.858884 (PMC9149596; doi:10.3389/fcell.2022.858884)
Supplement: Supplementary file 1 [file DataSheet1.docx]

**Supplementary Material**

**Covariation of Pluripotency Markers and Biomechanical Properties in Mouse Embryonic Stem Cells**

**Oliver Brookes^1^, Stephen D. Thorpe^1,2^, Olga Rigby Evans^1^, Michael C. Keeling^1^, David A. Lee^1*^**

^1^School of Engineering and Materials Science, Queen Mary University of London, London, United Kingdom

^2^UCD School of Medicine, UCD Conway Institute of Biomolecular and Biomedical Research, University College Dublin, Dublin, Ireland

*** Correspondence:**David A. Lee
[d.a.lee@qmul.ac.uk](mailto:d.a.lee@qmul.ac.uk)


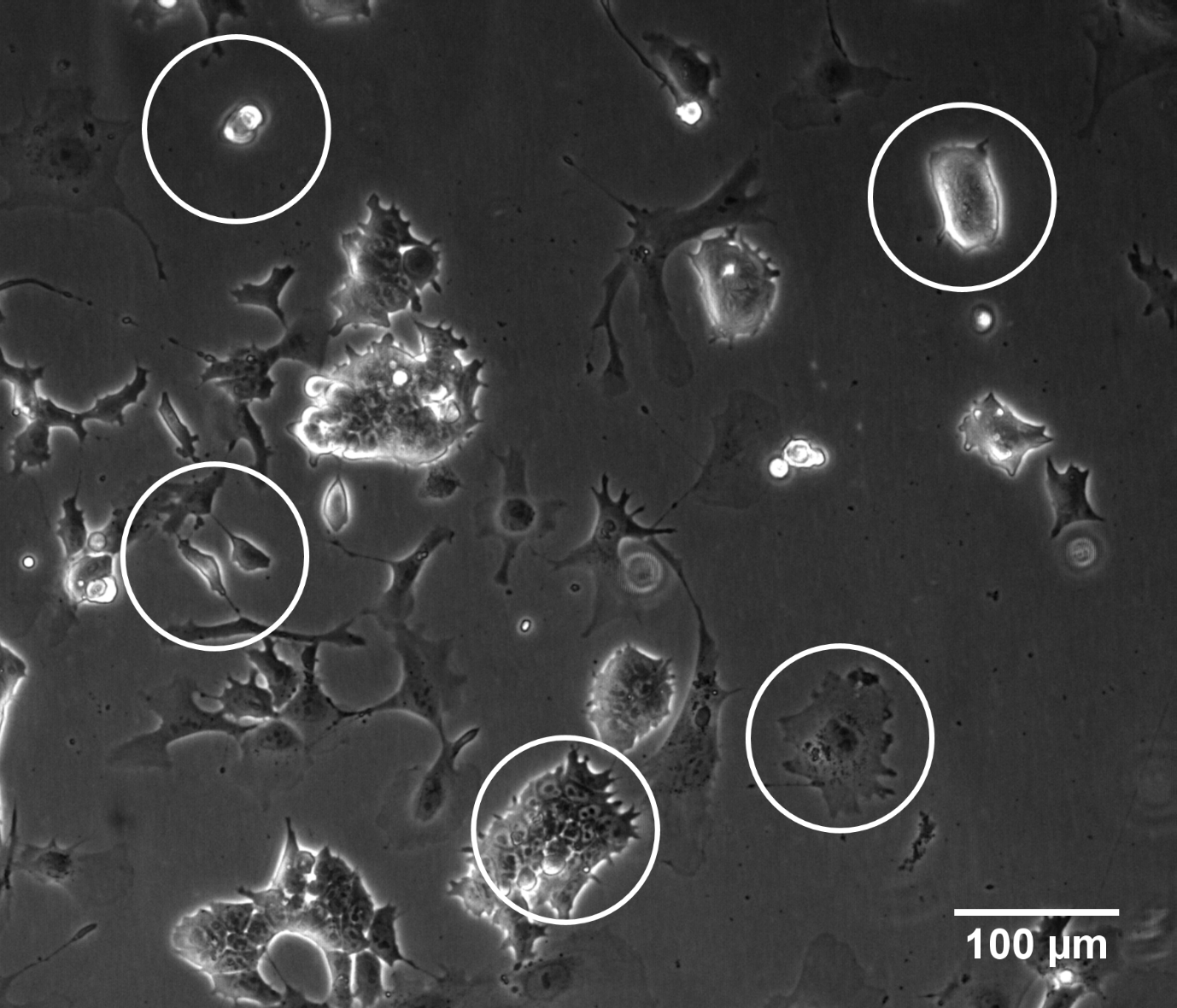


Supplementary Figure S1: Diverse morphologies can be found among mESCs cultured in LIF. Note that this image was selected because it shows many examples of diverse morphological characteristics and is not representative of the relative abundances of morphologies seen in routine culture.


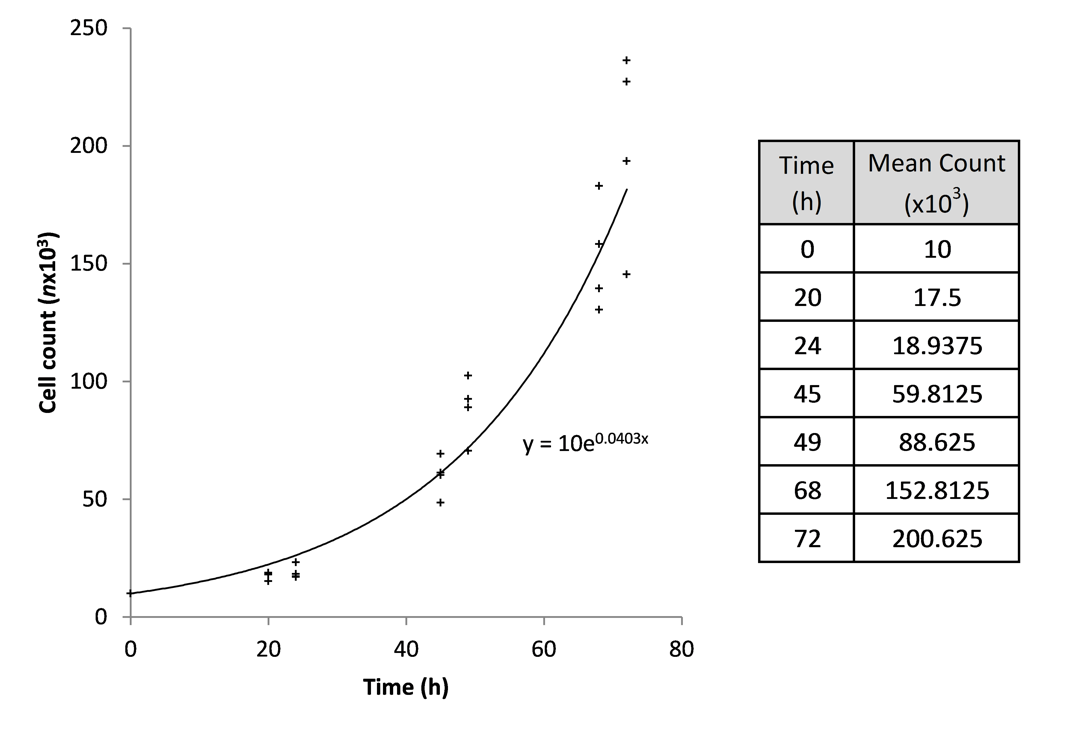


Supplementary Figure S2: mESC growth dynamics over 72 hours. Cell count presented against time and fit with an exponential function which provides a cell doubling time of 17.2 h. *n* = 4.


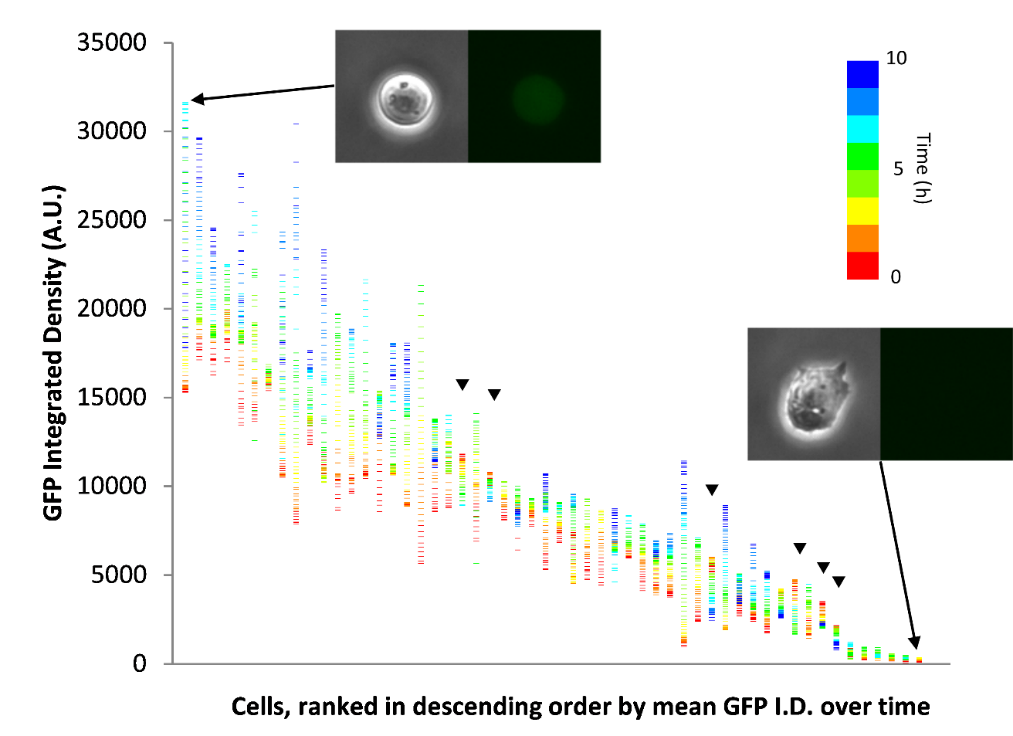

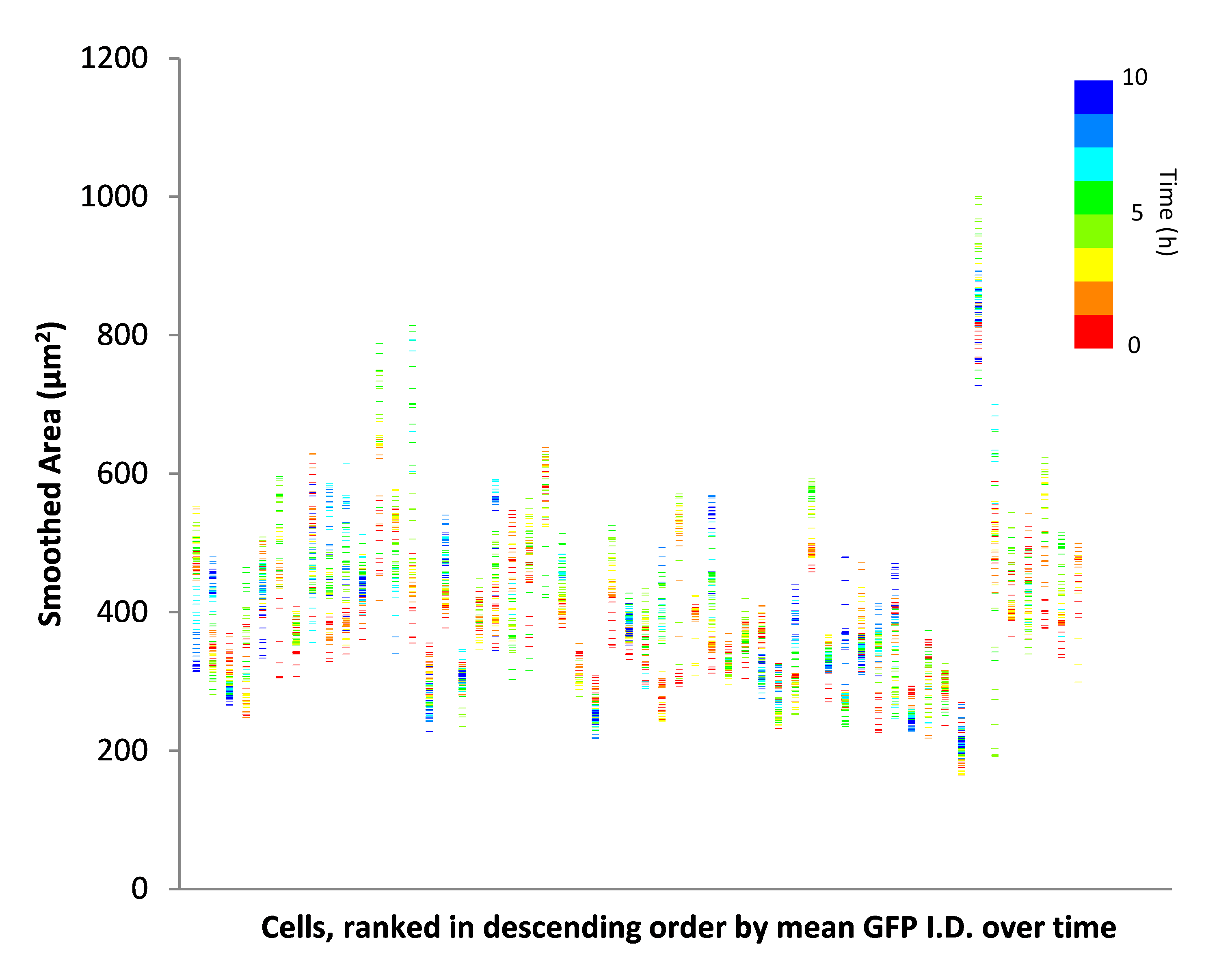


**B**

Supplementary Figure S3: Fluctuation in Rex1-GFP intensity over time is associated with mean Rex1-GFP intensity. (A) Rex1-GFP integrated density plotted per cell against the time averaged GFP integrated density over 10 h. Representative mESCs with highest and lowest time averaged Rex1-GFP intensity are shown as insets. Arrowheads indicate cells where Rex1-GFP integrated density decreased with time. (B) Smoothed cell area assessed from phase contrast images for mESCs over 10 h. Smoothing function is a 5 px Gaussian blur applied to permit robust automation of image segmentation. Smoothed area is plotted for each cell against time averaged GFP integrated density. *n* = 54 cells.


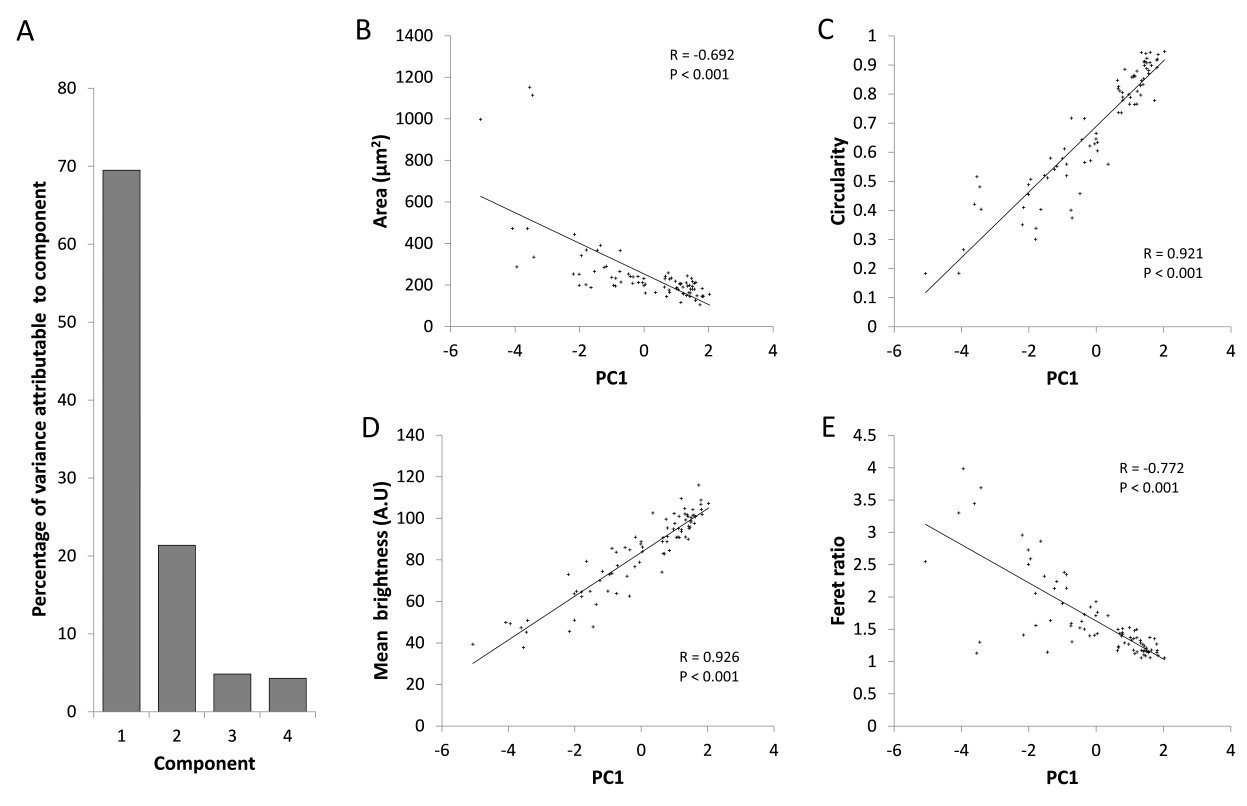


Supplementary Figure S4: Validation of the first principal component as a categorical descriptor of area, circularity, phase brightness and Feret ratio. Principal components analysis was carried out on morphological data obtained from phase contrast images of Rex1-GFPd2 cells cultured in LIF/FBS medium on gelatin. (A) The Scree plot shows that the first component accounts for 69.5% of the variance, components 1 and 2 collectively account for 90.9% of the variance. (B-E) All metrics show strong positive or negative correlations with the first principal component (PC1). A higher PC1 value is associated with smaller, brighter, rounder and less elongated cells. *n* = 85 cells. Pearson correlation test.


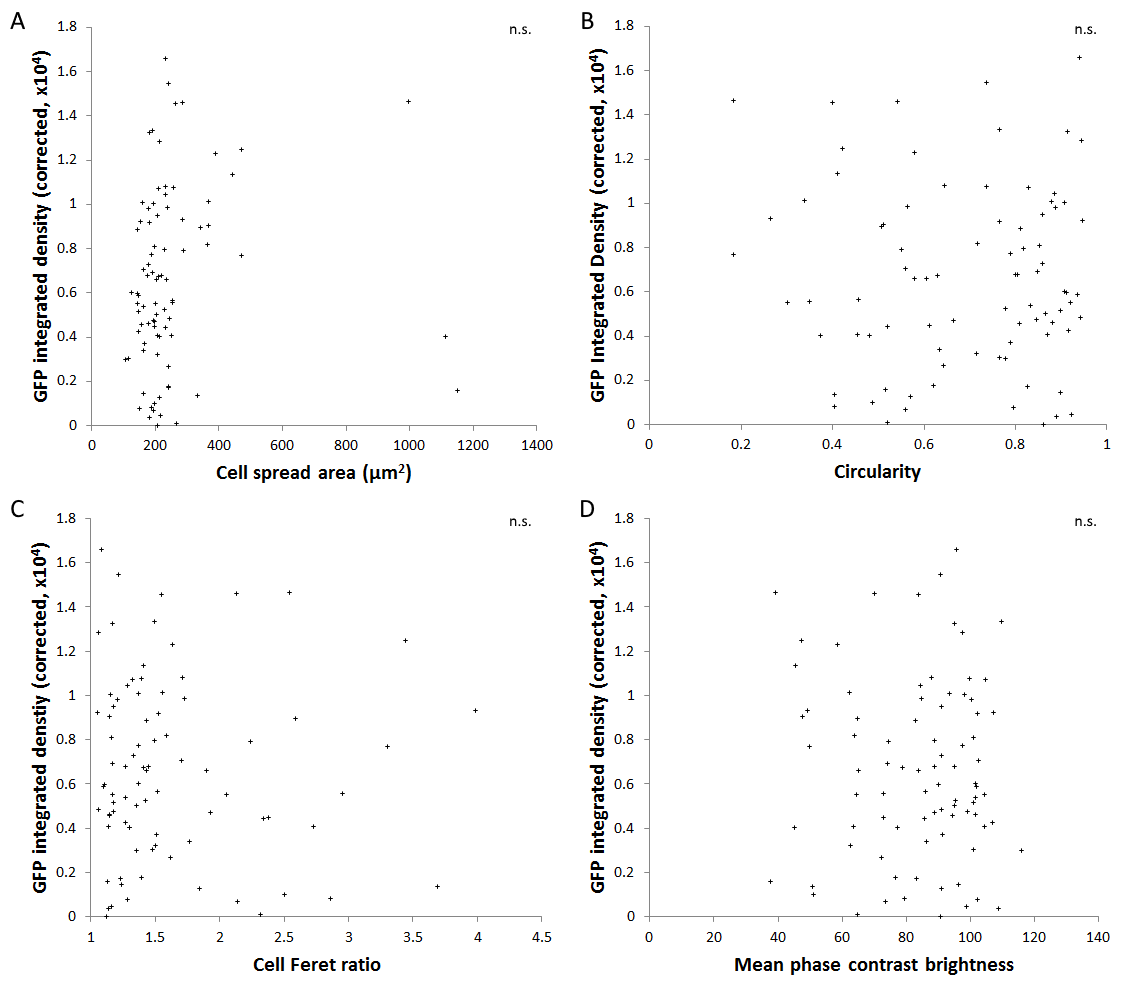


Supplementary Figure S5: Rex1-GFP expression does not correlate with cell morphology. Morphological descriptors of cells were derived from phase contrast images of Rex1-GFPd2 cells cultured on gelatin coated dishes for 6 h and plotted against Rex1-GFP integrated density. (A) Cell spread area, (B) circularity, (C) cell Feret ratio, and (D) mean phase contrast brightness. *n* = 84 cells, Pearson correlation test: n.s.: *p* > 0.05.
